# Stakeholder perspectives on clinical research related to therapies for rare diseases: therapeutic misconception and the value of research

**DOI:** 10.1186/s13023-020-01624-0

**Published:** 2021-01-12

**Authors:** Kylie Tingley, Doug Coyle, Ian D. Graham, Pranesh Chakraborty, Kumanan Wilson, Beth K. Potter, In collaboration with the Canadian Inherited Metabolic Diseases Research Network

**Affiliations:** 1 School of Epidemiology and Public Health, University of Ottawa, 600 Peter Morand Crescent, Ottawa, ON K1G 5Z3 Canada; 2 Ottawa Hospital Research Institute, Ottawa, ON Canada; 3 Metabolics and Newborn Screening, Department of Pediatrics, Children’s Hospital of Eastern Ontario, Ottawa, ON Canada; 4 Department of Pediatrics, University of Ottawa, Ottawa, ON Canada; 5 Newborn Screening Ontario, Ottawa, ON Canada; 6 Bruyère Research Institute, Ottawa, ON Canada; 7 Department of Medicine, University of Ottawa, Ottawa, ON Canada

**Keywords:** Rare diseases, Qualitative research, Research participation, Evidence-based medicine, Therapeutic misconception

## Abstract

**Background:**

For many rare diseases, few treatments are supported by strong evidence. Patients, family members, health care providers, and policy-makers thus have to consider whether to accept, recommend, or fund treatments with uncertain clinical effectiveness. They must also consider whether and how to contribute to clinical research that may involve receiving or providing the therapy being evaluated.

**Objective:**

To understand why and how patients and families with rare metabolic diseases, specialist metabolic physicians, and health policy advisors choose whether to participate in studies and how they use and value research.

**Methods:**

We conducted separate focus group interviews with each stakeholder group (three groups in total); two groups were conducted by telephone and the third was held in-person. Participants were recruited using purposive sampling. We analyzed each interview transcript sequentially using a qualitative description approach to inductively identify key themes. Several strategies to ensure credibility and trustworthiness were used including debriefing sessions after each focus group and having multiple team members review transcripts.

**Results:**

Four patients/caregivers, six physicians, and three policy advisors participated. Our findings did not support conventional perspectives that therapeutic misconception (gaining access to treatment) is the main motivating factor for patients/caregivers to participate in clinical research. Rather, patients’/caregivers’ expressed reasons for participating in research included advancing science for the next generation and having an opportunity to share their experiences. Patients/caregivers and physicians described the difficulties in weighing risks versus benefits of accepting treatments not well-supported by evidence. Physicians also reported feeling conflicted in their dual role as patient advisor/advocate and evaluator of the evidence. Policy advisors were primarily focused on critically appraising the evidence to make recommendations for the health system.

**Conclusions:**

Stakeholders differ in their perspectives on rare disease research but share concerns about the risks versus benefits of therapies when making individual- and population-level decisions.

## Background

For many rare diseases, there are few or no established treatments supported by strong evidence that the intervention alters the natural history of illness [1, 2]. This paucity of proven effective treatment options requires patients, caregivers, health care providers, and policy advisors to consider whether to accept, recommend, or fund emerging or controversial treatments where there is substantial uncertainty about potential benefits and harms, decisions which may be particularly difficult for diseases which are progressive and life-shortening [3–5]. Several authors have reported that patients and care providers demonstrate a willingness to try treatments without existing evidence because of a hoped-for or even expected clinical benefit over supportive care or no treatment [3, 4, 6–8]. This expectation of benefit conflicts with one of the central tenets of conducting research to establish the effectiveness of a new or existing intervention, which is the concept of *clinical equipoise* [9]*.*

Clinical equipoise, the presence of uncertainty or disagreement in the expert medical community about the comparative benefits or harms for a given intervention [10], provides the ethical basis for assigning study participants to different treatment groups in a clinical research study [9, 11]. Pai et al. reported that one of the barriers to conducting clinical research for rare diseases is the perceived lack of clinical equipoise among patients, caregivers, and health care providers [4]. If patients and/or care providers choose to participate in a trial to gain access to an intervention they already believe to be effective, then there is no clinical equipoise from their perspective [12]. This phenomenon is known as *therapeutic misconception,* which exists when clinical trial participants believe that the purpose of research is to benefit the individual rather than to generate generalizable knowledge to advance science [13, 14]*.* Therapeutic misconception leads to a reluctance among some patients and caregivers to participate in studies where there is a chance they will be assigned to a placebo or control group rather than the new treatment group [12, 15]. Coupled with small, geographically dispersed patient populations and characteristic clinical heterogeneity for many rare diseases [12, 16], this reluctance among patients and caregivers to participate may exacerbate challenges faced by researchers to recruit adequate sample sizes for rare disease clinical studies and thus limit the conclusions that can be drawn [17].

Recognizing that it is challenging to recruit a sufficient number of participants for clinical trials related to rare diseases, several alternative study designs have been proposed to make participation more attractive to patients and families by maximizing the time spent on- or guaranteeing the provision of- the experimental treatment [18, 19]. For example, in crossover and n-of-1 trials each participant receives both the control and experimental treatments, but the order in which each treatment is delivered is randomized [20, 21]. Using adaptive randomization procedures to reduce the likelihood of being assigned to the less effective treatment group over time may also make participation in clinical research studies more appealing [20, 22]. While these alternative designs may improve participant recruitment, there remains a need to consider how to reconcile the scientific and ethical concept of clinical equipoise with the potential therapeutic misconception experienced by patients or families when choosing to participate in clinical research.

As part of a larger project that seeks to develop specific guidance for the evaluation and synthesis of evidence for treatments for rare diseases, the objective of this paper is to describe different stakeholder perspectives on the role of research related to the development and evaluation of treatments for rare diseases. Specifically, we were interested in developing a deeper understanding of why and how patients and families, health care providers, and health policy advisors choose whether to participate in trials or other types of research, and how they use research findings to support decision-making at both an individual- and population- level.

## Methods

This study is described according to the Standards for Reporting Qualitative Research reporting guidelines [23].

### Qualitative approach

Recognizing that there were likely to be differing perspectives among stakeholders on the generation and evaluation of evidence for clinical interventions for rare diseases, we chose to conduct a series of focus group interviews to better understand the factors that stakeholders take into consideration when making decisions about research related to clinical interventions. Our study was conducted from an interpretivist point of view using a qualitative description approach, designed to allow researchers to develop a deep understanding of and describe a particular phenomenon based on participants’ experiential knowledge [24, 25].

### Sampling and recruitment strategy

We recruited participants using purposive sampling, a deliberate, non-probability sampling method used to select participants who can provide rich data related to the research topic [26, 27] comprising three groups of stakeholders: rare disease patients or caregivers, physicians, and policy advisors. Each group was chosen because they had formal knowledge and/or lived experience related to the research topic. To facilitate the focus group interviews, we chose rare inherited metabolic diseases (IMD) as a case study. For the patients and caregivers, we further narrowed the selection to those diagnosed with mucopolysaccharidoses (MPS), a group of IMD with several characteristics that typify many rare diseases (e.g., significant clinical heterogeneity; see Box 1 for description of MPS). Individuals were eligible to participate if they were adults diagnosed with MPS or were the adult caregiver (i.e., parent/guardian) of someone diagnosed with MPS, if they were a physician providing health care to those diagnosed with rare IMD, or if they had experience in evidence review activities that resulted in recommendations for the development, use, or reimbursement of interventions for rare diseases (policy advisors). Between five and eight individuals were sought for each focus group according to established focus group methodology in order to facilitate discussion while allowing each participant to be heard [26].

**Box 1:** Brief description of mucopolysaccharidoses

Mucopolysaccharidoses (MPS) are a group of seven heritable conditions that have an autosomal recessive inheritance pattern, except for MPS II which is X-linked [43]. Overall birth prevalence estimates for MPS vary by country/region and range from 1.04 to 4.8 per 100,000 live births [44]. These disorders are characterized by specific enzyme deficiencies that cause an accumulation of glycosaminoglycans (GAG) in the lysosomes of most cells [43, 45]. This buildup of GAG results in a wide spectrum of cell, tissue, and organ damage. The clinical manifestations of MPS begin early in life and are chronic, progressive, and typically involve multiple organ systems. Common clinical symptoms include: vision, hearing, cardiovascular, airway, and joint problems, organomegaly, musculoskeletal and facial abnormalities, among others [43, 45]. Similar to many other rare diseases, there is substantial clinical heterogeneity both between different MPS and within the same MPS [43, 45]. The spectrum ranges from mildly affected to severely affected. In addition to other symptoms, severely affected individuals may also experience neurocognitive deficits that are not present in more attenuated forms of the diseases [43, 45]. Currently, transformative treatments are limited for MPS, so care is generally supportive to help manage various symptoms [45]. For those with severe MPS I, early hematopoietic stem cell transplantation is recommended as standard of care to slow the progression of symptoms, particularly neurocognitive impairments [46, 47]. In addition, enzyme replacement therapy, an expensive orphan drug, is available for MPS I, II, and VI32,33; however, the extent of its efficacy is debated in the literature.	

We distributed recruitment invitations by email to physician members of the Garrod Association (a professional organization whose members are involved in caring for children with IMD), to policy advisors by a member of their professional network using publicly available contact information, and to patients/caregivers attending the Canadian MPS’s Society’s 2017 annual Family Meeting. Individuals interested in participating in the focus groups contacted the lead author (KT) for more information and to confirm eligibility. Eligible respondents were asked to provide signed informed consent to participate in the study.

### Data collection

Focus group were held separately for each stakeholder group; the patient and family focus group was conducted in person in conjunction with the Canadian MPS Society’s 2017 Annual Family Meeting, while focus groups with physicians and health policy advisors were conducted via teleconference. We conducted two focus groups by telephone in order to reach a geographically dispersed group of people across Canada who would otherwise be unable to gather in-person [28]. We reasoned physicians and policy advisors would likely have greater familiarity with the topic, thus may be comfortable convening via teleconference, whereas an in-person focus group may be important for patients and families.

We developed a semi-structured interview guide for each focus group that included questions related to the generation and evaluation of evidence for clinical interventions for rare diseases. Topics included: general perspectives on rare disease research, reasons for participating in research activities, outcomes used in clinical studies, and challenges in establishing treatment efficacy and effectiveness. The interview guide was reviewed by all members of the research team and by a representative from the Canadian MPS Society. One team member (KT) conducted all three focus group interviews, with at least one additional team member attending as an observer.

### Data processing and analysis

Each interview was audio-recorded with participants’ consent and transcribed for data analysis. The transcripts were analyzed sequentially using thematic analysis [29], which involved generating a set of initial codes based on interesting features of the data and then organizing those codes into key themes related to the research topic. To do this, a series of research team meetings were held to review the transcripts and inductively identify emerging concepts from each interview. Key concepts that were identified in the focus group data were organized into a coding system that was applied by one member of the study team (KT) using NVivo 10 software (QSR International Pty Ltd.) across the entire data set. Coded transcripts were reviewed and verified by a second team member (BP) to confirm all codes had been applied appropriately and that no themes had been overlooked. We used several strategies to ensure credibility and trustworthiness of our data [30], including: debriefing sessions after each focus group to identify key perspectives, multiple research team members reviewing transcripts, multiple team discussions to identify themes, and transcript coding verification by a second team member.

## Results

### Focus group characteristics

We completed three focus group interviews with a total of 13 participants (physicians n = 6; policy advisors n = 3; patients/caregivers n = 4). Each focus group interview lasted between 45 and 75 min. Across the three groups there were nine women and four men. Participants were from five provinces in Canada: British Columbia, Alberta, Ontario, Quebec, and Newfoundland and Labrador. Regardless of group size, all participants were engaged throughout the discussion with very little prompting from the moderator. Data from our focus group interviews revealed several key themes related to therapeutic misconception, reasons for participation in research activities, and how stakeholders value research.

### Making choices about participation in research

**Fig. 1:**
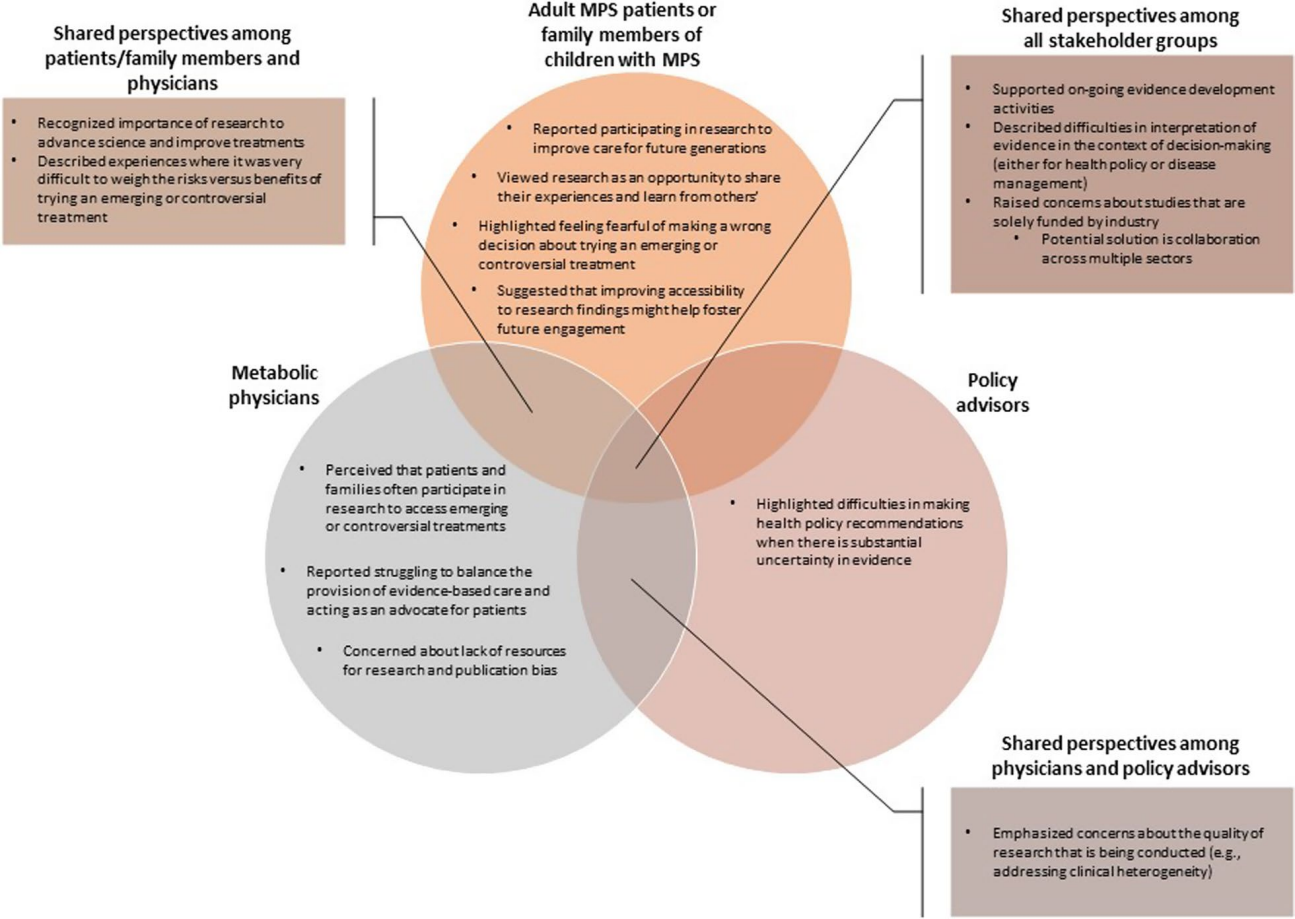
Shared and unique perspectives among focus group participants about participation in

Given their role in the health system, policy advisors are typically not directly involved in clinical research, so results related to participation in research are derived from focus groups with patients, caregivers, and physicians. Both patients/caregivers and physicians identified several reasons for participating in studies that seek to evaluate therapies for rare diseases. Some of their perspectives overlapped and others were unique to a single group (Fig. 1).

For example, some members of the physician group described their perception that patients and families often participate in clinical research, and even believe they are benefitting, out of a sense of desperation or willingness to try anything, especially when treatment options are limited.

“…you have patients or families that are experiencing a devastating condition and they want to try anything.” – Physician 4.

“… a lot of these families are extremely invested in being on this therapy because it’s their only therapeutic option.” – Physician 6.

Contrary to this view, patients and caregivers more frequently reported other reasons for choosing to participate in research activities and did not explicitly mention choosing to participate based on gaining access to treatment. For example, participants viewed involvement in research activities as a form of advancing science and an act of altruism of potential benefit to the next generation of individuals affected by the disease, understanding that they or their family members may be unlikely to personally benefit.

“…I think research of any kind is always useful for progressing science, and I’ve been involved in a number of different kinds of research studies over the years.” – Patient/caregiver 3.

“I find it’s hard because research takes so long [murmurs of agreement from the group]. It’s like, when you start off with this and talk about research and all this stuff, you have a lot of hope, as in wow it’s going to be done in no time, but then living with it, you think, you know what, maybe in somebody else’s lifetime, but it won’t be in our child’s lifetime. But you have to keep researching because if you stop, well it will be in nobody’s lifetime.” – Patient/caregiver 1.

“…participating in research is really important, especially with such a complex and ever-changing environment with rare diseases and particularly MPS. Yeah, so just for the learning and for sharing information.” – Patient/caregiver 4.

Patients and/or caregivers also described approaching research as an opportunity to share their own experiences and in turn to benefit from research findings that describe a broad range of experiences of other patients and families, to help inform decision-making.

“I think it’s important to have a lot of information because nobody has the same thoughts. I know from experience, your thoughts change from early diagnosis from like 13 years ago to now. I have a totally different way of thinking about the medication, the outcome, the whole thing.” – Patient/caregiver 1.

“I think it’s important because sometimes it all depends on the experience of what other people lived sometimes people tell you not to go there because they’ve had a bad experience. So, I like having the bad and the good ones too and then make up my mind and take better decisions.” – Patient/caregiver 2.

Some physician participants had similar views on the importance of research for furthering the development and improvement of rare disease therapies.

“…you’re advocating for your patient and if these treatments don’t ever happen, then they’ll never evolve, so that we won’t then get on to a better treatment. So, I think it’s extremely important that these treatments do develop, but it’s just tricky, sort of, coming up with the new treatments.” – Physician 4.

It was clear that not all patients and caregivers found it easy to make the decision to participate in a research study or to try a new therapy. Participants described being fearful of making the wrong decision in choosing whether to participate.

“For parents, it’s scary because if you say, okay we’ll try this, but what if you made the wrong decision [agreement from others]. That’s what always goes around in my head, what if I did it wrong? And usually we talk about it, my husband and I, but it seems to be like okay, mom you make the decision, but I don’t want to make it all by myself. What if I’m wrong?” – Patient/caregiver 1.

Patients and their caregivers also reported difficulty with weighing the risks versus benefits of trying a new therapy and spoke about uncertainty about whether it would be “worth it”.

“P1: But it’s scary to try other things. After one thing fails, it’s like okay, we could do this, but it sounds a little drastic. P4: I know, but what do we have to lose? P1: So what if it harms your child more than… P4: I know. P1: Sometimes you’re so afraid that you just don’t want to do anything. P4: Yup.” – Patient/caregiver 1 & 4.

“I think it depends too on what you have to do to get that little bit of change. Is it going to cost a lot of pain and anxiety and for just a little bit? It’s not really worth it because all that time spent could have been quality time spent doing, oh let’s go out and just sit in the sun…” – Patient/caregiver 1.

Similarly, one of the participants from the physician group described an experience of having to weigh risks versus benefits and wondering whether trying an experimental treatment was the right decision for their patients.

“I was involved with two babies with [severe inherited metabolic disease] quite early when [treatment] was just first available… and in retrospect, now that we have more knowledge about who is going to respond to [treatment], you know, I think it may have been that there was some harm done for those families, they could have had better quality of life. Both babies died, but you know, I think we were very optimistic at the time based on the publication of the studies, and you do wonder at the end of the day did I, was it more harmful for those families in regard to taking away quality time they could have spent with the baby and the family versus being back and forth in the hospital?” – Physician 4.

Despite the difficulties and uncertainty in making decisions to participate in clinical research or to try an experimental treatment, one caregiver highlighted the importance of being persistent and continuing to ask questions and do research.

“You always have to push. You can’t sit back and just accept things sometimes. It’s like, I know [name]’s situation came to a halt, but still kind of want to know why? And yeah, keep pushing and find out why does something happen? Why does it stop? And it’s always, you got to keep push, push, push, push. No matter who and how, you just have to keep asking questions.” – Patient/caregiver 1.

Lastly related to participation in research activities, patients or caregivers expressed a desire for research to be more accessible and noted that sharing findings from research in which they’ve directly participated may help encourage further research engagement.

“I find that when I read studies, I like the introduction and the conclusion, because the middle stuff is so scientific that even the ex-[profession] in me can’t understand it.” – Patient/caregiver 3.

“…user-friendly for the non-scientist parent. If it’s too overwhelming then you don’t really grasp. It could be sort of coded in a way that is easy for a tired parent.” – Patient/caregiver 4.

“…I think that’s a really important piece to keep people motivated to participate in these things is to at least have some sort of follow through that allows us to see if what we shared made any kind of a difference. So that would be one thing that I’d like to see…” – Patient/caregiver 4.

### Perspectives on the value of research

There was general agreement among participants in all three groups about the importance of supporting evidence development for new or existing therapies for rare diseases (Fig. 1). However, given their diverse backgrounds, there were differences across groups in how participants reported using research findings, the specific concerns they had with respect to the quality of clinical research, and the value of research to them in their roles as providers, evaluators, and users of care (Fig. 1).

For example, participants in both the physician and policy advisor groups were largely critical of the quality of research that is conducted for many rare disease therapies. They described difficulties in interpreting the available clinical evidence with respect to its value for informing decisions about real-world patient care, particularly for rare diseases characterized by important clinical heterogeneity.

“…this is a huge problem in terms of trying to interpret information from clinical studies, from treatment outcomes. How do we look at clinical trials versus actual clinical work that we do, which is much longer term than that of short clinical trials? How do we deal with some of the heterogeneity of the lysosomal storage diseases?” – Physician 5.

“And given the wide degree of heterogeneity with the drugs that we’re dealing with or the diseases that we’re dealing with, we know we’re going into this with a huge degree of uncertainty about whether or not there really is any evidence to support these therapies are going to work”. – Policy advisor 2.

Physician participants also reported that when considering how to use research findings, they struggled between providing care that is evidence-based, which requires a critical evaluation of research, and wanting to be an advocate for their patients, especially when their patients have limited treatment options.

“…most of the clinical trials are with few patients and are relatively short-term, so there’s always this conflict I think for clinicians between the short-term outcome measures and also our role as advocates for patients and families that may be very interested in pursuing these treatments, these very expensive treatments… you want to be an advocate but you [are] also a scientist and, you know, you may not be impressed by the outcomes or sometimes even study designs….” – Physician 4.

In considering how to best use evidence to inform routine patient care, one physician expressed concern about interpreting findings that show improved outcomes because of frequent interactions with the healthcare system as a result of being involved in a research study.

“One of the issues that’s been brought up by some of these studies as well is that when these patients are getting [treatment], they’re generally seeing the physician more frequently as well, and this is always a complicating factor in terms of treatment benefit. When they’re seeing the physician more frequently, they’re more likely to get interventions that are unrelated to the [treatment] because they’re there at that time and they’re complaining about X and Y, and they’re more likely to get treated earlier on, as opposed to patients who have had no therapy, and might only see the doctor one a year.” – Physician 1.

Another concern included potential publication bias in the context of rare diseases in that study designs that are conventionally considered low quality are more difficult to get published.

“The problem is that it’s often very difficult to get [observational] studies published, right? Even in journals dealing with rare disorders…” –Physician 1.

Finally, one concern that was raised in all three groups with respect to the quality of research was the difficulty of conducting high quality rare disease studies due to limited resources. In addition, participants across groups were concerned about potential bias in studies that are solely funded by pharmaceutical companies.

“And I think the conspiracy theorist in me wonders about who funds the research? Where is the funding coming from and what are their interests?” – Patient/caregiver 4.

“I have to say that there are concerns about the [name of research study] as well because, I mean, difficulties in obtaining funds continuously for a long period of time because it’s not pharma funded and also just because of the resources. …there’s still problems with that study because we just don’t have the manpower to properly run a study and design it properly etcetera.” – Physician 1.

“I think in a sense we are stuck with nobody having large enough pockets to fund a study looking at whether it’s [treatment] or whether it’s [treatment], we just don’t have the funding to do that without pharma. So, you’re between a rock and a hard place.” – Physician 5.

Some participants suggested that one way to overcome this would be to approach research in a more collaborative way that involves individuals from across sectors (e.g., health technology assessment agencies, health policy decision makers, and academic researchers as well as industry).

“…perhaps having partnerships between pharmaceutical companies, governments, and clinicians to ensure that we do get appropriate long-term follow-up is going to help us get that data and again, improve treatments.” – Physician 6.

## Discussion

We identified several themes from our focus group data related to why individuals choose to participate in research activities and how different stakeholders value and use research in their roles as evaluators, care providers, and recipients of care. Patients, caregivers, and physicians that participated in our focus group interviews shared the understanding that research is important for the development and improvement of treatments; however, physicians discussed concerns that rare disease patients and families often participate in clinical research studies because of potential therapeutic misconception and, as a result, patients may be unwilling to accept assignment to a placebo or comparator group. Previous discussions in the literature have also posited that patients and families choose to participate in clinical research studies because of desperation and that the expectation of personal benefit can threaten clinical equipoise [3, 4, 6, 7]. In contrast, patients and caregivers who contributed to our study had more nuanced views about participating in clinical research which extended beyond gaining access to a potential therapy, raising questions about the influence of therapeutic misconception in their decision-making about participating. Individuals in our study reported participating in clinical research studies to advance knowledge for future generations affected by the disease, to share personal experiences, and as an opportunity to learn from experiences of others. A survey of more than 3000 people affected by a rare disease conducted by the European Organization for Rare Disorders similarly found that the main motivation for respondents to participate in research was to help the community and make scientific advancements rather than gaining access to new treatment options [31]. These findings suggest patients and caregivers understand that participating in research has implications beyond accessing potential treatments and that those implications play a role in decisions about taking part.

Patients, caregivers and some physicians did speak extensively about the difficulty of weighing the personal benefits and risks, including opportunity costs to patients, of participating in a clinical study or accepting a treatment that is not well-supported by evidence. This finding indicates that these stakeholder groups do consider the individual implications of participating in research but, again in contrast to the therapeutic misconception, it suggests that they recognize that experimental therapies may carry personal risk or may not be effective. This perspective demonstrates that clinical equipoise may not be significantly compromised among this stakeholder group. While our work demonstrated that patients, caregivers, and health care providers valued research, it remains important to manage expectations of personal benefit and possible harms when strong evidence regarding an emerging intervention is unclear or not yet available.

Given their different roles in the health system as evaluators, care providers, and recipients of care, the ways in which participants from each group used and valued research findings differed. As described, patients, caregivers and physicians described potential personal benefits and risks of using treatments in the absence of strong evidence. Physicians also reported being conflicted in their role as both advocate/advisor for their patients and as an evaluator of the evidence base, suggesting that their priorities relate to whether there is sufficient evidence to support recommending a particular treatment and how to balance that evidence with patient and caregiver preferences. Policy advisor participants were primarily concerned about the quality of research that is being conducted. Policy advisors did not share the perspectives of patients or caregivers beyond the broad theme that uncertainty makes decision-making more difficult. This is perhaps not surprising given that the role of policy advisors is to critically appraise research and recommend if a treatment should be made available and/or reimbursed in the health system. Together, these findings suggest it is important to consider different roles and evidence needs among stakeholders when developing clinical research studies, approaching potential study participants, defining and prioritizing outcomes, and/or conducting evidence syntheses to ensure that scientific research is relevant and meaningful.

To our knowledge, there are few published studies discussing how different stakeholders value and engage with research in the context of rare diseases. Kesselheim and colleagues conducted a focus group interview study with patients, caregivers, and patient advocates; some similar themes emerged from their data including that participants reported experiencing difficulties in weighing the potential risks and benefits of accepting a controversial therapy. Some findings conflicted with the results from our study, namely our patient and caregiver participants did not report feeling desperate to try anything and did not discuss feeling uncomfortable in participating in a study with the potential to be randomized to a control group [32]. A focus group study with parents and clinicians of individuals with Duchenne muscular dystrophy regarding participation in research touched on similar themes of parents and clinicians finding it difficult to make decisions to participate in research when there is such a limited evidence base [33]. Regarding the themes we identified concerning the quality of research, it is established in the literature that the small, geographically-dispersed, and clinically heterogeneous patient populations that typify many rare diseases present challenges for meeting conventional evidence standards for establishing treatment effectiveness (e.g., there are few or no randomized controlled trials for many rare disease therapies) [18, 34].

While there is little published empirical research regarding stakeholder attitudes toward participation in rare disease clinical research, there is a rich literature discussing motivations for participating in research in other disease areas. For example, Dupont and colleagues discuss ethical aspects of the participation of pediatric cancer patients in clinical trials [35]. Similar to our findings, the authors highlight that there is a complex risk–benefit judgment entailed in families’ decision-making about whether to participate in clinical research and that patient and family preferences should be carefully considered [35]. While there are key differences between pediatric cancers and rare genetic diseases (e.g., cancers are defined by incidence whereas rare genetic diseases are defined by prevalence, disease course may be very different), the challenges for clinical research are similar in many ways, especially given the relatively low incidence of pediatric cancer and the uncertainties surrounding potential therapeutic options [36, 37].

Though it was not discussed extensively by the participants in our study, some literature has shown therapeutic misconception as a strong motivating factor when choosing to participate in research [13, 14, 38, 39]. For example, Hendersen et al. demonstrated in their study of early phase gene therapy that while some participants may understand they are in a research study to generate generalizable knowledge, they may still have unrealistic expectations about the direct benefit of the therapy under study [38]. A recent survey of health care professionals working in pediatric cancer centres also demonstrated similar results to ours in that the majority of health care professionals surveyed believed perception of medical benefit for the child was a primary motivating factor for parents’ consent for participation in early-phase clinical trials [40]. Therapeutic misconception is a complex concept that warrants further investigation in the field of rare diseases.

An important strength of our study is the in-depth insights generated about participating in clinical research studies and the perceived value of research in supporting health care decision-making. Using focus group methodology allowed participants to compare and contrast their perspectives with those described by others in the group and to build on others’ ideas. Another strength of our study is the use of multiple methods to ensure credibility and trustworthiness of our data [30]. While the preferred sample size for focus group interviews is between five and eight individuals, we only recruited three and four participants to the policy advisor and patient/caregiver focus groups, respectively. In addition, conducting two focus group interviews via teleconference may have reduced the interactions between participants and in turn, limited the richness of our data set [28]. Despite the small number of participants in these groups and the inclusion of telephone focus group interviews, there was still a lively discussion in each group. The small number of people in each group also allowed each participant ample time to speak and fostered a very in-depth discussion of the research topic. Thus, we do not feel that these factors substantially reduced the richness of the data collected. In future focus group studies, adding a webinar option could provide a good alternative to in-person focus groups [41]. We did not formally assess data saturation, so it is possible that we have not identified an exhaustive set of themes; however, the literature suggests that three focus group interviews is enough to identify the most prevalent themes in a dataset and approximately 80% of all possible themes [42]. Transferability of the results to the broader rare disease community may be limited given that we recruited a purposive sample of metabolic physicians and MPS patients and caregivers. In addition, patients and caregivers were recruited from a list of attendees at a national meeting for MPS patients and families which may have restricted the variation in perspectives. However, inherited metabolic diseases and specifically MPS, do exemplify some of the typical characteristics of rare diseases, including small patient populations, significant clinical heterogeneity, and few available treatment options; thus, it is likely that many of the themes identified in this work could be transferable to other rare diseases. Future work could explore perspectives among different rare disease groups to understand if their views align with our study. Additional work among other rare disease groups would also enable a more in-depth investigation of how disease progression/severity, timing, and other factors affect decision making regarding participation in research (e.g., is there less willingness to participate in clinical research studies among patients/families if the manifestations of the disease are less severe?).

## Conclusion

In the context of rare diseases, health care decision-making (individual- and population- level) and clinical research are often intertwined, and the lines between individual care and participation in clinical studies are sometimes blurred. Our study identified different perspectives on how diverse stakeholders choose to participate in and use research in their roles as health care users, care providers, and policy advisors. Notably, the conventional wisdom that patients and family members participate in clinical research studies because of therapeutic misconception was not supported. We believe there is an opportunity to further investigate this finding in other rare disease populations and assess whether there is heterogeneity in opinions held among rare disease stakeholders from other jurisdictions as well. Overall, we found that stakeholders differ in their perspectives on rare disease research but share concerns about the risks versus benefits of therapies when making individual- and population-level decisions. This shared perspective provides opportunities for engaging all stakeholders toward collaborative approaches to the design, conduct, and use of research to evaluate care. Developing a deeper understanding of why stakeholders choose to participate in research activities and how they value research will inform the design of future clinical research studies and ensure that results are meaningful.

## Data Availability

In order to protect the privacy of our study participants, research data are not publicly available.
